# Impulsive-compulsive behaviour in early Parkinson’s disease is determined by apathy and dopamine receptor D3 polymorphism

**DOI:** 10.1038/s41531-023-00596-9

**Published:** 2023-11-15

**Authors:** Hendrik Theis, Stéphane Prange, Gérard N. Bischof, Merle C. Hoenig, Marc Tittgemeyer, Lars Timmermann, Gereon R. Fink, Alexander Drzezga, Carsten Eggers, Thilo van Eimeren

**Affiliations:** 1 Faculty of Medicine and University Hospital Cologne, Department of Nuclear Medicine, Multimodal Neuroimaging Group, University of Cologne, 50937 Cologne, Germany; 2 Faculty of Medicine and University Hospital Cologne, Department of Neurology, University of Cologne, 50937 Cologne, Germany; 3 Université de Lyon, CNRS, UMR 5229, Institut des Sciences Cognitives Marc Jeannerod, Lyon, 69500 France; 4 Forschungszentrum Jülich, Institute for Neuroscience and Medicine (INM-2), Molecular Organization of the Brain, 52428 Jülich, Germany; 5 Max Planck Institute for Metabolism Research, 50931 Cologne, Germany; 6 Cologne Excellence Cluster on Cellular Stress Responses in Aging-Associated Diseases (CECAD), University of Cologne, 50931 Cologne, Germany; 7 Faculty of Medicine and University Hospital Marburg, Department of Neurology, University of Marburg, 35043 Marburg, Germany; 8 Forschungszentrum Jülich, Institute of Neuroscience and Medicine (INM-3), Cognitive Neuroscience, 52428 Jülich, Germany; 9 German Center for Neurodegenerative Diseases (DZNE), 53127 Bonn-Cologne, Germany; 10 Department of Neurology, Knappschaftskrankenhaus Bottrop, 46242 Bottrop, Germany

**Keywords:** Parkinson's disease, Risk factors, Neurodegeneration

## Abstract

Impulsive-compulsive behaviour (ICB) is a frequently observed non-motor symptom in early Parkinson’s disease after initiating dopamine replacement therapy. At the opposite end of the motivated behaviour spectrum, apathy occurs in early Parkinson’s disease even before dopamine replacement is started. The co-occurrence of these behavioural conditions in Parkinson’s disease raises questions about their relationship and underlying pathophysiological determinants. In previous imaging or genetic studies, both conditions have been associated with the limbic dopaminergic system. The risk variant of the Ser9Gly polymorphism of the dopamine receptor D3 (DRD3) is linked to increased dopamine affinity in the limbic striatum. With this in mind, we investigated how ICB expression is explained by apathy and DRD3 polymorphisms and their effects on grey matter volume and dopamine synthesis capacity. Fifty-four patients with early Parkinson’s disease took part in anatomical T1-weighted MRI. Forty of them also underwent dynamic PET imaging using [18F]DOPA to measure striatal dopamine synthesis capacity. Further, Ser9Gly (rs6280) gene polymorphism influencing the DRD3 dopamine-binding affinity was determined in all patients. The severity of impulsive-compulsive behaviour and apathy was assessed using the Questionnaire for Impulsive-Compulsive Disorders Rating Scale and the Apathy Evaluation Scale. ICB and the severity of apathy were indeed positively correlated. Apathy and the DRD3 polymorphism were interactive risk factors for ICB severity. Apathy was significantly linked to atrophy of the bilateral putamen. Patients with the DRD3 risk type had reduced dopamine synthesis capacity in the putamen and limbic striatum, apathy was associated with reduced dopamine synthesis capacity in the limbic striatum. The results of [18F]DOPA reached only trend significance. Apathy in drug-naïve PD patients might be a consequence of impaired striatal dopaminergic tone. This may represent a predisposing factor for the development of ICB after the initiation of dopamine replacement therapy. The risk type of DRD3 could further amplify this predisposition due to its higher affinity to dopamine.

## Introduction

Impulsive-compulsive behaviours (ICB) are among the most disabling and frequent non-motor symptoms in patients with early Parkinson’s disease (PD)^1,2^. In particular, ICB also comprises subsyndromal impulsive-compulsive behaviour and not only severely manifest impulse control disorders (ICD), such as pathological gambling, compulsive shopping, binge eating, and hypersexuality. ICB symptoms are an extreme burden for patients and their surroundings. Therefore, identifying risk factors for ICB and understanding its pathophysiology is critical for the development of treatment strategies.

We previously conceptualised a *vulnerability-stress* model for the development of ICB in PD. In this model, *vulnerability* is underpinned by a hypodopaminergic state in the striatum, which could be genetically modified, while *stress* is induced by dopamine replacement therapy^3^.

The Ser9Gly single nucleotide polymorphism (rs6280) of the dopamine receptor D3 (DRD3) constitutes a candidate for a genetic risk modifier (vulnerability), since the serine (Ser) to glycine (Gly) substitution increases dopamine-binding affinity^4^. The DRD3 receptor is primarily located in the limbic part of the striatum and primarily acts as a presynaptic autoreceptor inhibiting tonic dopamine release^5^. Therefore, as previously described in ICB^6^, a reduced dopamine synthesis capacity in the limbic striatum would dovetail with a higher affinity for dopamine. Indeed, carriers of the risk allele are more prone to develop ICB. Moreover, the DRD3 risk variant is associated with impulsive decision-making in the general population^7,8^.

Another behavioural marker for altered dopaminergic function is the presence of apathy. Apathy is a very frequent non-motor symptom in early PD, but unlike ICB, often predates the initiation of dopamine replacement therapy^9^. Recent findings indicate that apathy and ICB co-occur in many patients with PD^10,11^. Apathy is a significant part of dopamine agonist withdrawal syndrome (DAWS), which occurs in patients with ICB^12^. Besides, patients with preoperative ICB are prone to develop apathy after subthalamic nucleus deep brain stimulation (STN-DBS), while those with preoperative apathy are at risk of developing ICB after surgery^13^. Apathy in PD seems to be associated with striatal atrophy^14^ and a reduction of dopamine transporter (DAT) density^15^ in the striatum. Indeed, patients with ICB also showed a reduced DAT density in the ventral striatum^16,17^.

Therefore, apathy might be a specific risk factor for developing ICB when dopamine replacement therapy is initiated.

We hypothesised that apathy and the DRD3 polymorphism might be synergistic determinants for ICB severity. The aim of the current study was to investigate the impact of these determinants on the phenotypic expression of ICB. Furthermore, we were interested in the structural and functional imaging differences linked to these determinants in terms of grey matter volume and dopamine synthesis capacity. We hypothesised that carriers of DRD3 risk variants would show a relatively reduced dopamine synthesis capacity in the limbic striatum since DRD3 receptors are primarily located in the limbic striatum, and ICB is linked to reduced dopaminergic signalling in this area^6^. We expected that apathy is linked to atrophy and reduced dopamine synthesis capacity in the striatum.

## Results

### Link between severity of apathy and ICB

**Fig. 1:**
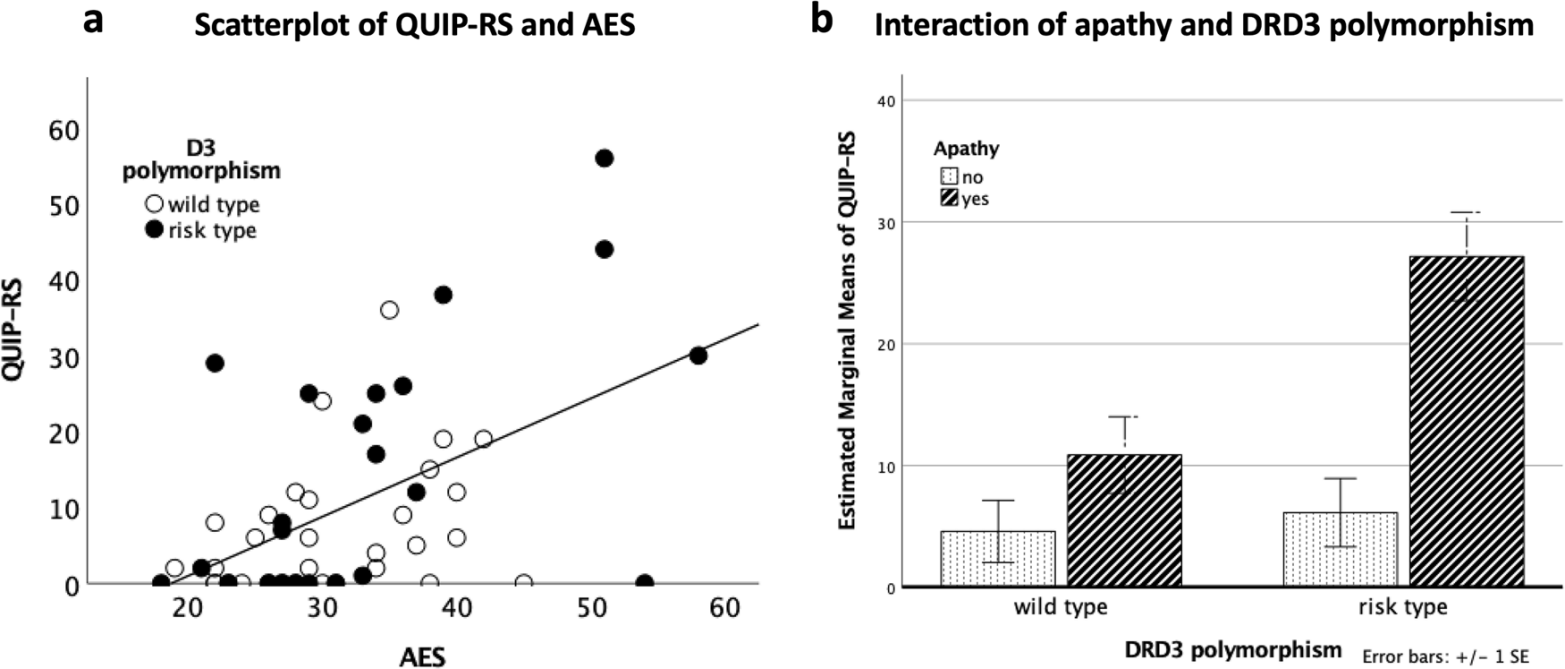
The link between ICB, apathy and DRD3 polymorphism. **a** Significant positive correlation between AES and QUIP-RS. **b** Bar diagram of the interaction analysis of DRD3 polymorphism and apathy status. The error bars refer to the standard error of the mean.

There was a significant positive correlation between QUIP-RS and AES (*rho* = 0.446, *P* < 0.001) (Fig. 1a), which means that patients with more severe apathy had more severe ICB. Four patients had an AES score higher than 50. The correlation analysis remained significant even after the removal of these patients (rho = 0.406, *p* = 0.003). In the healthy controls, there was no significant correlation between the severity of apathy and ICB (rho = −0.244, *p* = 0.381). In patients, severity of apathy and depression were strongly correlated (rho = 0.600, *p* < 0.001), but we did not find an effect of severity of depression on ICB that was independent of apathy severity by using a partial rank correlation (rho = 0.148, *p* = 0.291).

### Distribution of ICB, apathy and DRD3 polymorphism

**Fig. 2:**
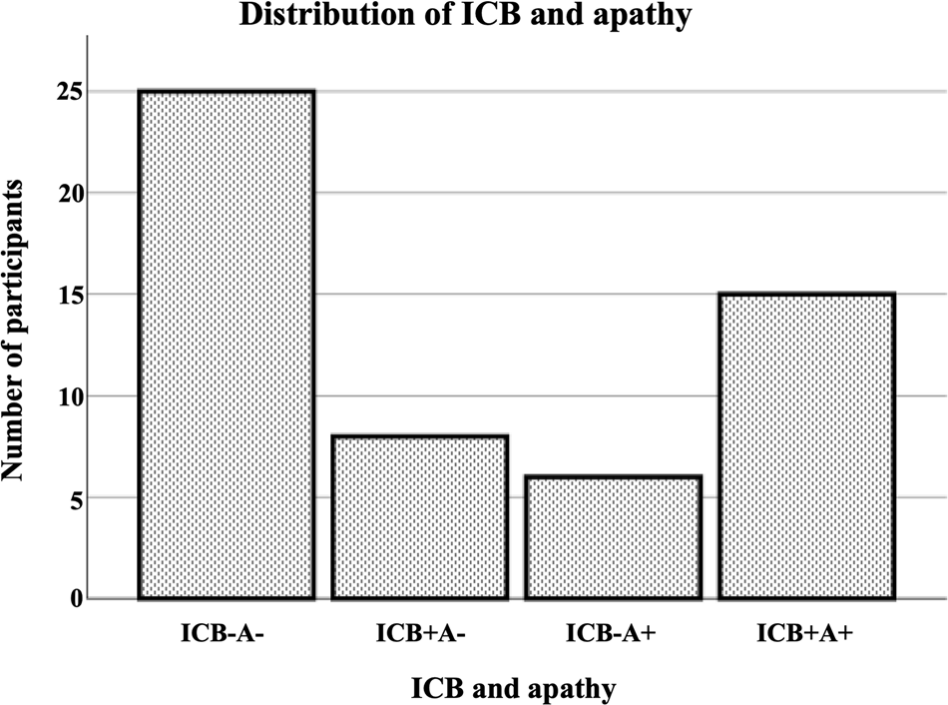
Distribution of apathy and ICB in our cohort. No ICB and no apathy (ICB−A−). ICB and no apathy, (ICB+A−), no ICB and apathy (ICB−A+), ICB and apathy (ICB+A+).

The distribution of ICB, apathy and DRD3 polymorphism was as follows: 23 patients presented at least one ICB and 15 of them had multiple ICBs. The distribution among the different ICB categories was as follows: 2 pathological gambling, 9 hypersexuality, 3 excessive buying, 8 binge eating, 14 hobbyism, 12 punding and 17 dopamine dysregulation syndrome. 21 patients scored positive for apathy. 15 patients reported apathy and ICB. Figure 2 shows the distribution of apathy and ICB in our cohort. Genotyping for DRD3 resulted in 30 carriers of DRD3− (wild type) and 24 carriers of DRD3+ (risk type). Among the healthy controls, 5 subjects suffered from ICB and 3 of them from multiple ICBs. ICB categories were as follows: 0 pathological gambling, 6 hypersexuality, 2 excessive buying, 3 binge eating, 3 hobbyism, 1 punding and 0 dopamine dysregulation syndrome. The healthy controls did not fulfil the criteria for apathy. DRD3 polymorphism was not assessed in controls. Therefore, the ANOVA and imaging analyses could not be replicated in the healthy controls.

### ANOVA of apathy status and DRD3 polymorphism

The model was statistically significant (*F*(6,48) = 13.14, *P* < 0.001). The main effects of apathy (*F*(1, 48) = 20.13, *P* < 0.001, BCa 95% CI [−32.28, −8.657]) and of DRD3 polymorphism (*F*(1, 48) = 8.33, *P* = 0.006, BCa 95% CI [14.79, 57.19]) significantly determined QUIP-RS. The interaction of DRD3 polymorphism and apathy status (*F*(1, 48) = 5.79, *P* = 0.02, BCa 95% CI [.536, 27.93]) also significantly determined QUIP-RS (see Fig. 1b).

### Comparisons between DRD3− and DRD3+ and Apathy− and Apathy+

Group comparisons between carriers of DRD3− and DRD3+ as well as Apathy− and Apathy+ are presented in Table 1. Patients with apathy showed more severe symptoms of ICB, impulsivity (BIS) and depression. Additionally, motor complications were more severe in A+ than in A-. Nine patients had an UPDRS IV higher than 4 indicating at least moderate motor complications, eight of them were apathetic^18^.

**Table 1:** Group comparison between DRD3 wild type and DRD3 risk type as well as non-apathetic and apathetic PD patients.

	All patients	Apathy			DRD3 polymorphism		
		No	Yes	*p* value	Wild type	Risk type	*p* value
Number of subjects	54	33	21	∅	30	24	∅
Age	70 (13)	70 (15)	70 (10)	0.993	70.5 (13)	68.5 (11)	0.280
Male	35	21	14	0.82	19	16	0.799
Right-handed	50	31	19	0.443	28	22	0.496
AES	30 (11)	**27 (7)**	**38 (8)**	**<0.001**	29.5 (13	30 (11)	0.807
QUIP-RS	6 (18)	**1 (8)**	**15 (24)**	**<0.001**	4.5 (11)	7.5 (26)	0.297
PANDA	23 (7)	23 (6)	22 (8)	0.593	23.5 (9)	23 (6)	0.523
MMSE	29 (2)	29 (2)	28 (3)	0.165	29 (2)	29 (2)	0.726
BDI-II	8 (9)	**6 (6)**	**14 (9)**	**<0.001**	8 (9)	7 (9)	0.402
BIS	59 (9)	**56 (7)**	**62 (10)**	**<0.001**	60 (7)	58 (11)	0.438
UPDRS III (off)	26 (11)	25 (12)	26 (11)	0.186	26 (10)	24 (14)	0.644
UPDRS IV	2 (4)	**0 (2)**	**4 (4)**	**<0.001**	2 (4)	1 (3)	0.170
LED (mg)	430 (354)	425 (325)	450 (376)	0.936	453.5 (304)	400 (462.5)	0.365
Intake of DA-agonists	46	29	17	0.697	24	22	0.277
DA-LED (mg)	100 (142)	120 (125)	80 (110)	0.101	100 (152.5)	150 (122.5)	0.294
Disease duration	4 (5)	4 (5)	3 (5)	0.675	4 (5)	3 (6)	0.285

Median and interquartile range (in brackets). *P* values based on Mann–Whitney *U* test or Fisher’s exact test. The median and interquartile range for the complete group were added for better readability. *UPDRS III* Unified Parkinson’s Disease Rating Scale part 3, *UPDRS IV,* Unified Parkinson’s Disease Rating Scale part 4, *QUIP-RS* Questionnaire for Impulsive-Compulsive Disorders in Parkinson’s Disease Rating Scale, *AES* Apathy Evaluation Scale, *BDI-II* Beck Depression Inventory version 2, *BIS* Barratt Impulsiveness Scale, *MMSE* Mini-Mental State Examination, *PANDA* Parkinson Neuropsychometric Dementia Assessment, *LED* Levodopa equivalent dose, *DA-LED* dopamine agonist equivalent dose.

Significant values in bold.

### Imaging—VBM

**Fig. 3:**
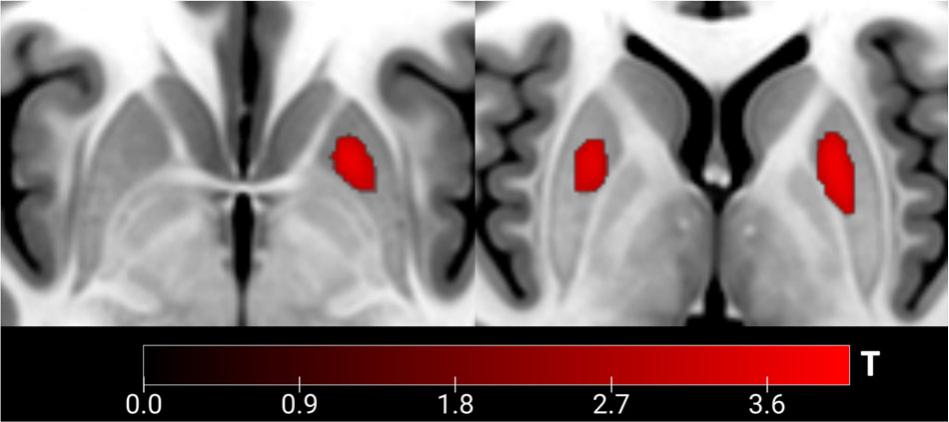
The link between apathy and grey matter volume. Significant negative effect of apathy on grey matter volume in bilateral putamen. Axial view.

There was a significant negative effect of apathy on grey matter volume in the left (left: *x* = −22, *y* = 8, *z* = −2, pseudo-*t* = 4.09) and right putamen (*x* = 24, *y* = 6, *z* = −2, pseudo-*t* = 3.62). The results survived FWE correction on the cluster level. The DRD3 polymorphism was not associated with any volumetric differences in the striatum. See Fig. 3.

### Imaging—[18F]DOPA-PET

The following results are only trend significant and reported with a liberal threshold of *p* < 0.001 uncorrected. The risk type of the DRD3 polymorphism was associated with a reduced dopamine synthesis capacity in the right limbic striatum (*x* = 16, *y* = 20, *z* = −4, pseudo-*t* = 3.11), and bilateral putamen (right: *x* = 28, *y* = 12, *z* = −2, pseudo-*t* = 3.45; left: *x* = −28, *y* = −6, *z* = 0, pseudo-*t* = 2.8). Apathy was linked to a reduced dopamine synthesis capacity in the right limbic striatum (*x* = 10, *y* = 20, *z* = −4, pseudo-*t* = 3.23).

## Discussion

We found that apathy and risk type of DRD3 polymorphism are interactive risk factors for the severity of ICB. Apathy was linked to significant atrophy of the putamen, while the risk type of the DRD3 polymorphism was not associated with any volumetric differences. PET imaging revealed at trend significance that the DRD3 risk type and apathy had a negative effect on dopamine synthesis capacity in the limbic striatum and putamen.

Consistent with previous work^10^, apathy and ICB co-occurred in our early-stage PD cohort to a similar extent. In a cross-sectional study with 887 PD patients, 61% of the patients with ICB symptoms also reported apathy, whereas 41.3% of the apathetic PD patients also reported ICB^11^. A study with a large population sample of young adults showed that the severity of apathy and impulsivity were positively correlated^19^. We could replicate this finding in a group of patients with PD. Given this positive correlation, we speculate that apathy might constitute a risk factor for ICB, based on a striatal vulnerability and its modulation by dopaminergic replacement therapy. It is worth noticing that apathetic patients scored higher on the BIS. A previous study demonstrated that impulsivity measured by BIS did not lead to a higher prevalence of ICB in PD, but increased their severity^20^. It is worth speculating that impulsivity goes along with similar imaging changes like ICB, i.e., a reduction of dopaminergic terminals to the ventral striatum^16^. Notably, this seemingly counterintuitive behavioural co-expression of ICB and apathy may result from patients experiencing increased sensitivity for immediate reward while experiencing difficulties in pursuing long-term goals due to apathy and discounting^21^. In controls, we could not find a correlation between the severity of ICB and apathy. Controls with ICB did not suffer from apathy. It is tempting to speculate that this link between apathy and ICB might be more specific for PD. Interestingly, apathy was associated with more severe motor complications. This is in line with the fact that apathy predicted motor complications in PD patients in a previous study^22^. However, against our expectations, dopamine dysregulation syndrome was not associated with motor complications. Furthermore, there was no effect of depression on ICB that was independent of severity of apathy. Therefore, we assume that apathy and depression are similar sides of the same hypodopaminergic “coin”.

We report that apathy was linked to atrophy of the bilateral putamen and this effect was independent of the DRD3 polymorphism. This region is associated with action-reward association learning and the storage of motor memories emphasising the link between apathy and ICB. Our finding goes along with striatal atrophy in non-demented apathetic PD patients^14,23^. In addition, apathetic elderly without PD also had a reduced volume of the putamen^24^. Apathy was not only associated with atrophy but was also linked to microstructural alterations in the anterior striatum in de novo PD patients^25^. We therefore speculate that—independent of the development of PD—apathy is causally linked with anatomical changes in the putamen.

We further found that the dopamine synthesis rate in the limbic striatum was reduced in patients with apathy independent of the DRD3 polymorphism. However, these results only reached trend significance, because they did not survive FWE correction. Therefore, they have to be interpreted with caution. This finding is in line with a previous raclopride study revealing that apathetic PD patients had a significantly reduced dopamine release in the entire striatum^26^. Our result is further supported by the fact that DAT density in the striatum was reduced in apathetic PD patients in a previous study^15^. Furthermore, apathetic PD patients demonstrated, compared to controls, a widespread dopaminergic and serotonergic degeneration in the caudate, putamen, and limbic striatum^27,28^. A study on PD patients with deep brain stimulation further emphasises a reduced function of the limbic striatum in apathy: A reduced preoperative glucose metabolism in the limbic striatum was associated with an increase in apathy after surgery^29^. We conclude that atrophy and reduced dopaminergic signalling in the striatum may promote the development of apathy. For further reading, we recently highlighted the role of the limbic striatum in PD in a systematic review^30^.

Interestingly, our behavioural results demonstrate that the severity of ICB increases together with apathy. However, not only apathy, but also the risk type of DRD3 polymorphism led to a more pronounced ICB phenotype. DRD3 are G-protein coupled receptors belonging to the DRD2-like family of dopamine receptors. Activation of these receptors inhibits adenylate cyclase and reduces cyclic adenosine monophosphate (cAMP) levels, which may affect dopamine synthesis directly. DRD3 is mainly found in the limbic striatum and the ventral tegmental area (VTA), where they act primarily as autoreceptors, resulting in negative feedback on presynaptic dopamine signalling, enhanced for risk type with a higher affinity for dopamine^7,31,32^. Here, we observed the trend that carriers of the risk allele have reduced dopaminergic synthesis capacity in the (limbic) striatum independent of apathy on a trend level. We conclude that this reduction in dopamine levels might be due to the enhanced affinity of those presynaptic receptors to dopamine and dopamine agonists^7^. Furthermore, we previously demonstrated that the severity of ICB was associated with a reduced dopamine synthesis capacity in the limbic striatum^6^. Taken together, these studies suggest that the DRD3 risk variant might be an important modulator to develop ICB via influencing the limbic dopaminergic tone.

Additionally, the postsynaptic effects of DRD3 in the limbic striatum could also contribute to the severity of ICB. A pharmacological fMRI design showed that dopamine agonists prevent pauses in dopamine transmission, thus limiting learning from negative feedback^33^. This effect might be amplified in carriers of the DRD3 risk type, since it has a higher affinity for dopamine and maybe also for dopamine agonists^7^. As a result of this tonic overstimulation of the postsynaptic membrane, dips in dopaminergic signalling might not be registered anymore, leading to a reduced sensitivity to negative consequences of actions. Note that a neuropathological study demonstrated that DRD3 levels in the limbic striatum were associated with ICB, with downregulation of the dopamine D3 receptor levels possibly occurring either as a consequence of the degenerative process or as a pre-morbid trait^34^.

To our knowledge, no other study could show the interactive effect of apathy and the DRD3 risk type on the severity of ICB. Indeed, apathy and the DRD3 risk type were associated with impaired function and structure of the striatum. The results of this study follow and extend the recently proposed vulnerability-stress model for ICB^3^. We speculate that apathy, especially in drug-naïve PD patients, is based on impaired function of the striatum. These apathetic patients might be especially vulnerable for ICB with the initiation of dopamine replacement therapy. This effect is amplified in patients with the risk type of DRD3 due to higher affinity to dopamine and especially dopamine agonists.

There are several limitations to consider. QUIP-RS and AES scores may reflect symptom severity over a relatively long time. Therefore, we cannot claim that symptoms of apathy and ICB actually co-occurred simultaneously. Effects of DRD3 risk variant on dopamine synthesis analysis only reached trend significance in this nonparametric permutation-based approach and should therefore be interpreted with caution due to the high rate of Type I errors. In our group comparisons of DRD3 wild type and risk type carriers did not reach significance for ICB severity in a Mann-Whitney-U-Test. However, in the ANOVA a significant independent effect of DRD3 polymorphism on ICB severity was found. Overall, apathy seems to play a stronger role for ICB severity than DRD3 type. Only fifteen healthy controls were tested for ICB and apathy. Unfortunately, DRD3 polymorphism was not assessed in controls. Here, we focused on dopaminergic systems. However, other neurotransmitters, such as serotonin, may play a critical role in developing apathy or ICB^35^. Further, within the dopaminergic system, other genes may also be associated with the development of ICB, e.g., DRD1 (rs4680), DAT (rs28363170), or COMT (rs4680)^36^.

In sum, our findings suggest that apathy and the DRD3 risk variant interact to confer a synergistic risk for the severity of ICB. Both of these factors were associated with impaired striatal function, which may contribute to the development and maintenance of ICB. Our results have important clinical implications, as they highlight the need for personalised treatment approaches that take into account both genetic and behavioural factors in the management of ICB.

Furthermore, our study adds to a growing body of literature demonstrating the complex interplay between genetic and environmental factors in the development of psychiatric disorders. Future research should explore the potential mechanisms underlying the interaction between apathy and the DRD3 risk variant, as well as the role of other genetic and environmental factors in the pathogenesis of ICB.

Finally, our findings also have broader implications for our understanding of the neurobiology of motivation and reward processing. The striatum plays a key role in these processes, and its dysfunction has been implicated in a range of psychiatric disorders. Our study highlights the importance of considering the complex interactions between different factors that contribute to striatal function and provides further evidence for the need to develop personalised treatment approaches that target the underlying neurobiology of neuro-psychiatric disorders.

## Methods

### Study participants

Participants were recruited as part of the German KFO-219 cohort^6,37,38^. We included only those patients who underwent genotyping and MRI imaging, resulting in fifty-four patients with early Parkinson’s disease and fifteen age-matched healthy controls. A subgroup of 40 patients also underwent [18F]DOPA-PET imaging. Patients were recruited from the Department of Neurology of the University Hospital of Cologne, with the diagnosis of idiopathic PD according to the UK Brain Bank Criteria^39^. Exclusion criteria were any other diseases that may affect brain function (e.g., stroke, tumour), dementia, and any issues regarding MRI safety. Dopamine replacement therapy was discontinued for 12 hours (levodopa) and 72 hours (for dopamine agonists) prior to PET scanning. The study was carried out following the International Ethical Guidelines and the Declaration of Helsinki. The ethics committee of the medical faculty of the University of Cologne (Germany) approved the study (application number: 12-265). Written informed consent was obtained from all participants.

### Clinical and neuropsychological data

We used the validated German version of QUIP-RS to assess ICB severity^40^. The Apathy Evaluation Scale (AES) was used to capture apathy severity^41^. These self-reported scores were obtained simultaneously. The Mini-Mental State Examination (MMSE) and Parkinson neuropsychometric dementia assessment (PANDA) evaluated cognitive functions. Furthermore, we assessed the Beck Depression Inventory (BDI-II) and the Barratt Impulsiveness Scale (BIS). The severity of motor symptoms in the unmedicated condition was assessed using the Unified Parkinson’s Disease Rating Scale Part III (UPDRS III). Motor complications were assessed with the Unified Parkinson’s Disease Rating Scale Part IV (UPDRS IV). Dopamine replacement therapy dosages were converted to levodopa equivalent daily doses and dopamine agonist equivalent doses, respectively^42^. The number of patients taking dopamine agonists was assessed.

### Genetic analyses

QIAamp DNA Blood Mini Kit (#51106, QIAGEN) was used to isolate DNA from a peripheral blood sample. For the assessment of DNA quality and concentration, an ND-1000 UV/Vis-Spectrophotometer (Peqlab) was used. For SNP genotyping of rs6280, 20 ng of DNA was analysed in triplicates using allelic discrimination assays (TaqMan SNP Genotyping Assays, Applied Biosystems by Invitrogen). Genotyping PCR was performed with a 7900HT Fast Real-Time PCR System (Applied Biosystems). Sequence Detection Software version 2.3 (Applied Biosystems) was used to process fluorescence data^37^. Groups were defined according to carriers of the DRD3 [DRD3− (wild type): T/T; DRD3+ (risk type): C/T, C/C].

### Image acquisition and preprocessing—MRI

54 PD patients underwent MRI imaging. Scanning was done on a 3 T Magnetom Prisma (Siemens, Erlangen). For the anatomical T_1_-weighted MPRAGE acquisition parameters were as follows: repetition time = 2300 ms, echo time = 2.32 ms, flip angle = 8, field of view = 230 mm, slice thickness = 0.9 mm, voxel size = 0.9 × 0.9 × 0.9 mm, number of slices = 192. In order to detect anatomical differences, we calculated structural volume based on voxel-based morphometry with the CAT12 toolbox (https://neuro-jena.github.io/cat/). The T1-weighted images were segmented automatically into grey and white matter. The grey matter volumes were smoothed by an 8 × 8 × 8 FWHM Gaussian Kernel and the total intracranial volume (TIV) was calculated.

### Image acquisition and preprocessing—[18F]DOPA-PET

Dynamic PET images were acquired in 3D mode with a 24-detector ring scanner ECAT EXACT HRRT (Siemens, Erlangen) over 90 minutes. The subjects were lying in a quiet and dim room during image acquisition. All patients received 100 mg of Carbidopa 1 hour before intravenous injection of [18F]DOPA, with an average activity of 185 MBq. A 10-min transmission scan with a retractable [68Ga]/[68Ge] ring source was performed to correct tissue attenuation. PET images were corrected for random coincidences, attenuation, and scatter. We normalised the images using the [18F]DOPA-PET template in SPM^43^. The voxel-wise net influx rate constant of [18F]DOPA (DOPA-K_i_), indicating dopamine synthesis capacity, was determined by a Patlak plot^6^.

### Statistical analyses—behavioural and genetic data

The distribution of normality for QUIP-RS and AES was tested using the Kolmogorov-Smirnow-Test in addition to visual inspection. In a first step, we examined the relationship between the severity of apathy and ICB. Therefore, we performed a Spearman correlation analysis with apathy and ICB as quantitative variables, i.e., QUIP-RS and AES. We performed this analysis in PD patients and healthy controls, separately. Additionally, we investigated how far the severity of depression had an effect on ICB that was independent of apathy. To do this, we performed a partial rank correlation with QUIP-RS and BDI-II controlling for AES.

In a second step, we examined how many patients of our cohort reached the established cut-offs for ICB and apathy. Positivity for ICB was defined according to previously established QUIP-RS cut-offs for the different ICB categories^40^. Positivity for apathy was defined by using a previously defined cut-off of the AES in PD (AES > 33)^41^.

In a third step, a two-way ANOVA was used to examine the interactive effect of apathy status (categorical variable) and DRD3 receptor polymorphism (categorical variable) on ICB severity (QUIP-RS, quantitative variable). Age and sex were included as covariates of no interest in this model. Using 1000 samples and the bias corrected and accelerated method (BCa), bootstrapped confidence intervals were calculated due to non-normal distribution of QUIP-RS.

In a final step, group comparisons of neuropsychological and demographic data between DRD3− (wild type) and DRD3+ (risk type) as well as apathetic and non-apathetic were calculated based on Mann-Whitney-U-Tests. For differences in the distribution of biological sex, handedness and intake of dopamine agonists, we applied Fisher’s exact test between the groups. Furthermore, we added a column summarising the demographical information of the entire group.

### Statistical analyses—imaging data

Apathy status and DRD3 polymorphism significantly predicted the severity of ICB. We performed a post-hoc analysis examining the independent effect of these two categorical predictors on striatal grey matter volume and dopamine synthesis capacity as neural correlates of the propensity to develop ICB. We tested how far grey matter volume and dopamine synthesis capacity were driven by the apathy status or DRD3 polymorphism and thereby led to more severe ICB as shown by the behavioural interaction. Therefore, we used multiple linear regression models. Due to the small sample size, different group sizes and nonparametric distribution of behavioural data, we performed voxel-based statistical nonparametric mapping using the SnPM13 package (http://www.nisox.org/Software/SnPM13/). Analyses were performed based on 5000 random permutations, and we applied variance smoothing with a 10 × 10 × 10 kernel. The Striatal Connectivity Atlas was used as an explicit mask (https://fsl.fmrib.ox.ac.uk/fsl/fslwiki/Atlases/striatumconn). A statistical threshold of *p* < 0.05 family-wise error (FWE)-corrected was considered significant. *P* values below *p* < 0.001 uncorrected were considered as trend significant. For image display, we used MRIcroGL (https://www.nitrc.org/projects/mricrogl).

For VBM, we specified a multiple linear regression model with apathy status (categorical variable), DRD3 carrier status (categorical variable) and TIV as covariates. There was no relevant collinearity with TIV and any other parameter of interest. In this model, the negative contrast of each factor was calculated. The other factors mentioned above were then set as covariates of no interest.

For [18F]DOPA-PET, a multiple linear regression model with DRD3 carrier status (categorical variable) and apathy status (categorical variable) as covariates was specified. In this model, the negative contrast of each factor was calculated. The other factors mentioned above were then set as covariates of no interest

### Reporting summary

Further information on research design is available in the Nature Research Reporting Summary linked to this article.

### Supplementary information

Strobe

Reporting summary

## Data Availability

The data that support the findings of this study are not openly available due to reasons of sensitivity but are available from the corresponding author upon request.

## Code availability

No code was generated during the study.
