# Supplementary material for: Impulsive-compulsive behaviour in early Parkinson’s disease is determined by apathy and dopamine receptor D3 polymorphism
Source: NPJ Parkinsons Dis. 2023 Nov 15;9:154. doi: 10.1038/s41531-023-00596-9 (PMC10651866; doi:10.1038/s41531-023-00596-9)
Supplement: Supplementary file 2 — Reporting summary [file 41531_2023_596_MOESM2_ESM.pdf]

nature portfolio

Corresponding author(s): Thilo van Eimeren

Last updated by author(s): 12.09.2023

Reporting Summary

Nature Portfolio wishes to improve the reproducibility of the work that we publish. This form provides structure for consistency and transparency in reporting. For further information on Nature Portfolio policies, see our Editorial Policies and the Editorial Policy Checklist.

Statistics

For all statistical analyses, confirm that the following items are present in the figure legend, table legend, main text, or Methods section.

n/a	Confirmed	
☐	☒	The exact sample size (*n*) for each experimental group/condition, given as a discrete number and unit of measurement
☐	☒	A statement on whether measurements were taken from distinct samples or whether the same sample was measured repeatedly
☐	☒	The statistical test(s) used AND whether they are one- or two-sided<br>*Only common tests should be described solely by name; describe more complex techniques in the Methods section.*
☐	☒	A description of all covariates tested
☐	☒	A description of any assumptions or corrections, such as tests of normality and adjustment for multiple comparisons
☐	☒	A full description of the statistical parameters including central tendency (e.g. means) or other basic estimates (e.g. regression coefficient) AND variation (e.g. standard deviation) or associated estimates of uncertainty (e.g. confidence intervals)
☐	☒	For null hypothesis testing, the test statistic (e.g. *F*, *t*, *r*) with confidence intervals, effect sizes, degrees of freedom and *P* value noted<br>*Give P values as exact values whenever suitable.*
☒	☐	For Bayesian analysis, information on the choice of priors and Markov chain Monte Carlo settings
☒	☐	For hierarchical and complex designs, identification of the appropriate level for tests and full reporting of outcomes
☒	☐	Estimates of effect sizes (e.g. Cohen's *d*, Pearson's *r*), indicating how they were calculated

Our web collection on statistics for biologists contains articles on many of the points above.

Software and code

Policy information about availability of computer code

Data collection	No commercial , open source or custom code was used to collect data in this study.
Data analysis	There was no commercial, open source or custom code generated for data analysis. SPM12 and SPSS Version 28.0.1.0 was used.

For manuscripts utilizing custom algorithms or software that are central to the research but not yet described in published literature, software must be made available to editors and reviewers. We strongly encourage code deposition in a community repository (e.g. GitHub). See the Nature Portfolio guidelines for submitting code & software for further information.

Data

Policy information about availability of data

All manuscripts must include a data availability statement. This statement should provide the following information, where applicable:

- Accession codes, unique identifiers, or web links for publicly available datasets
- A description of any restrictions on data availability
- For clinical datasets or third party data, please ensure that the statement adheres to our policy

Data will be made available upon reasonable request.

Research involving human participants, their data, or biological material

Policy information about studies with human participants or human data. See also policy information about sex, gender (identity/presentation), and sexual orientation and race, ethnicity and racism.

Reporting on sex and gender	Sex was assessed in the study design. There were no differences in terms of sex between the groups.
Reporting on race, ethnicity, or other socially relevant groupings	Race and ethnicity were not assessed.
Population characteristics	Mean age and sex was assessed. Concerning the PD diagnosis all information are given in Table 1.
Recruitment	Patients were recruited as part of the German KFO-219 cohort
Ethics oversight	medical faculty of the University of Cologne (Germany)

Note that full information on the approval of the study protocol must also be provided in the manuscript.

Field-specific reporting

Please select the one below that is the best fit for your research. If you are not sure, read the appropriate sections before making your selection.

☒ Life sciences ☐ Behavioural & social sciences ☐ Ecological, evolutionary & environmental sciences

For a reference copy of the document with all sections, see nature.com/documents/nr-reporting-summary-flat.pdf

Life sciences study design

All studies must disclose on these points even when the disclosure is negative.

Sample size	54 patients, 15 controls.
Data exclusions	No data was excluded
Replication	N/A
Randomization	N/A
Blinding	N/A

Reporting for specific materials, systems and methods

We require information from authors about some types of materials, experimental systems and methods used in many studies. Here, indicate whether each material, system or method listed is relevant to your study. If you are not sure if a list item applies to your research, read the appropriate section before selecting a response.

Materials & experimental systems

n/a	Involved in the study	
☒	☐	Antibodies
☒	☐	Eukaryotic cell lines
☒	☐	Palaeontology and archaeology
☒	☐	Animals and other organisms
☒	☐	Clinical data
☒	☐	Dual use research of concern
☒	☐	Plants

Methods

n/a	Involved in the study	
☒	☐	ChIP-seq
☒	☐	Flow cytometry
☐	☒	MRI-based neuroimaging

Magnetic resonance imaging

Experimental design

Design type	N/A

Design specifications	N/A
Behavioral performance measures	N/A

Acquisition

Imaging type(s)	Structural
Field strength	3T
Sequence & imaging parameters	T1-weighted MPRAGE acquisition parameters were as follows: repetition time = 2300 ms, echo time = 2.32 ms, flip angle = 8, field of view = 230 mm, slice thickness = 0.9 mm, voxel size = 0.9 x 0.9 x 0.9 mm, number of slices = 192.
Area of acquisition	Whole Brain

Diffusion MRI ☐ Used ☒ Not used

Preprocessing

Preprocessing software	SPM 12, CAT 12
Normalization	T1 images are normalized to a template space and segmented into gray matter (GM), white matter (WM), and cerebrospinal fluid (CSF).
Normalization template	TPM template of SPM
Noise and artifact removal	N/A
Volume censoring	N/A

Statistical modeling & inference

Model type and settings	Multiple linear regression
Effect(s) tested	*Define precise effect in terms of the task or stimulus conditions instead of psychological concepts and indicate whether ANOVA or factorial designs were used.*

Specify type of analysis: ☒ Whole brain ☐ ROI-based ☐ Both

Statistic type for inference (See Eklund et al. 2016)	cluster wise inference p<0.05
Correction	FWE

Models & analysis

n/a	Involved in the study
☒	☐ Functional and/or effective connectivity
☒	☐ Graph analysis
☒	☐ Multivariate modeling or predictive analysis
